# Genome-wide DNA methylation and hydroxymethylation analysis reveal human menstrual blood-derived stem cells inhibit hepatocellular carcinoma growth through oncogenic pathway suppression via regulating 5-hmC in enhancer elements

**DOI:** 10.1186/s13287-019-1243-8

**Published:** 2019-05-31

**Authors:** Yichen Wu, Xin Chen, Yongjia Zhao, Yanling Wang, Yifei Li, Charlie Xiang

**Affiliations:** State Key Laboratory for Diagnosis and Treatment of Infectious Diseases, Collaborative Innovation Center for Diagnosis and Treatment of Infectious Diseases, The First Affiliated Hospital, College of Medicine, Zhejiang University, Hangzhou, China

**Keywords:** Mesenchymal stem cell, HCC, Epigenetics, 5-Hydroxymethylcytosine, Enhancer

## Abstract

**Background:**

Epigenetic alteration is an important indicator of crosstalk between cancer cells and surrounding microenvironment components including mesenchymal stem cells (MSC). Human menstrual blood-derived stem cells (MenSCs) are novel source of MSCs which exert suppressive effects on cancers via multiple components of microenvironmental paracrine signaling. However, whether MenSCs play a crucial role in the epigenetic regulation of cancer cells remains unknown.

**Methods:**

Epigenetic alterations of hepatocellular carcinoma (HCC) mediated by MenSCs were examined by immunofluorescence, ELISA, and RT-PCR assays. The suppressive impact of MenSCs on HCC was investigated in vitro using CCK8, apoptosis, wound healing, and invasion assays and in vivo using a xenograft mice model. MeDIP-seq, hMeDIP-seq, and RNA-seq were used to identify the genome-wide pattern of DNA methylation and hydroxymethylation in HCC cells after MenSC therapy.

**Results:**

We show that HCC cells display distinct genome-wide alterations in DNA hydroxymethylation and methylation after MenSC therapy. MenSCs exert an inhibitory effect on HCC growth via regulating 5-hmC and 5-mC abundance in the regulatory regions of oncogenic pathways including PI3K/AKT and MAPK signaling, especially in enhancers and promoters. FOXO3 expression is rescued via reversal of 5-hmC and 5-mC levels in its enhancers and contributes to the activation of downstream apoptosis. Inactivation of the MAPK pathway further disrupts c-myc-mediated epithelial-mesenchymal transitions (EMT). Additionally, chemotherapy resistance-associated genes including ID4 and HMGA1 are suppressed via amending 5-hmC and 5-mC abundance at their regulatory regions. HMGA1 and BYSL might be potential targets for gene-modified MSC therapy.

**Conclusions:**

Our results confirm that MSCs could regulate the epigenetic mechanism of HCC cells and provide a novel concept for a modified MSC strategy or combination therapy with chemotherapeutics based on epigenetics.

**Electronic supplementary material:**

The online version of this article (10.1186/s13287-019-1243-8) contains supplementary material, which is available to authorized users.

## Background

Tumor malignant transformation is driven by a long-term gradual accumulation of genetic insults and epigenetic abnormalities [1, 2]. The dynamic plasticity of the epigenome in cancers closely correlates to microenvironmental cues, including infection [3], chronic inflammation [4], and hypoxia [5]. DNA methylation is one of the widely studied epigenetic modifications and plays an important role in diverse cellular processes, including genomic imprinting, embryonic development, and tumorigenesis [6–8]. The balance of DNA methylation in the genome is principally maintained by tet methylcytosine dioxygenases (TETs), which mediate demethylation, and DNA methyltransferases (DNMTs), which mediate methylation [9]. The stepwise accumulation of a DNA methylation burden in promoter CpG islands has been conclusively demonstrated to contribute to suppressor inactivation [10]. Active demethylation in mammals is mediated by the TET family, including TET1, TET2, and TET3, which oxidize 5-mC to 5-hydroxymethylcytosine (5-hmC), and further to 5-formylcytosine (5-fC) and 5-carboxylcytosine (5-caC) [11]. 5-hmC, a vital demethylation intermediate, is frequently diminished in tumorigenesis. Because 5-hmC is enriched in regulatory regions, changes in its distribution usually involve variation in transcriptional expression [12]. In addition, 5-hmC abundance in the genome adjusts dynamically in response to altered environmental stimuli [13, 14]. Hence, 5-hmC has been recommended as a sensitive marker for indicating cell burden [15, 16].

MSCs are adult pluripotent progenitor cells of multiple mesenchymal lineages that are widely studied in degenerative and inflammatory disorders [17]. Given their inflammation- and tumor-homing properties, MSCs have been considered as potential vehicles for cancer therapy that are able to target the sites of even microscopic tumors. Recently, strategies involving gene-modified MSCs that carried TRAIL [18], IL-12 [19], and an oncolytic virus [20] have been developed to improve their therapeutic effects. However, the clinical feasibility of MSCs for cancer therapy is hampered by a lack of sufficient understanding of the mechanisms of interaction between MSCs and cancer cells. Hence, a great deal of research is still needed to explore the role of MSCs in the regulation of cancer microenvironments. MenSCs, a novel type of MSCs, exhibit high proliferation, hypoimmunogenicity, and multiple differentiation potential [21, 22]. Unlike the MSCs isolated from the bone marrow, adipose, and umbilical cord, MenSCs are very easily obtained, being isolated from human menstrual fluid without pain, invasive procedures, or ethical issues. Previous studies have demonstrated the potential of MenSC-based therapy in different disease models, including liver fibrosis, fulminant hepatic failure, and acute lung injury [23–25]. As a cancer therapy, MenSCs have been shown to block the growth of ovarian cancer [26]. However, the potential of MenSCs to act on other cancers remains unknown.

Hepatocellular carcinoma (HCC) is one of the cancers in which pathogenic processes intimately correlate with epigenetic dysregulation under long-term burdens of infection and chronic inflammation. Previously, gene-modified MSCs applied to acute, chronic liver disease and even precancerous lesions have been shown to have a significantly beneficial effect. However, the role of MSCs in the epigenetic regulation of HCC is still unknown.

In the present study, we aimed to investigate whether MenSCs affect epigenetic regulation in HCC and exert anticancer effects via specific epigenetic alterations. Based on the multi-platform integration, we provided important evidence of distinct 5-hmC and 5-mC landscapes in HCC cells after MenSC therapy. Then, we showed that MenSCs exerted an anticancer effect via suppressing oncogenic pathways and reviving tumor suppressors through altering the abundance and distribution of 5-hmC and 5-mC in regulatory regions, especially enhancers. Our study not only contributes to demonstrate the epigenetic mechanism between HCC and microenvironment, but also suggests MenSCs would be promising vehicles for a modified MSC strategy based on epigenetics or combination therapy with chemotherapeutics in HCC.

## Materials and methods

### Cell culture

Menstrual blood was obtained from healthy female volunteers after informed consent, and MenSC isolation was approved by the Ethics Committee of Zhejiang University. MenSCs were isolated as previously described [27]. Briefly, menstrual blood samples were collected with DivaCups (Kitchener, ON, Canada) from volunteers during menstruation. The mononuclear cells were separated via density gradient centrifugation with Ficoll-Paque (Fisher Scientific, USA). The interlayer cells were collected carefully and cultured in Chang Medium (S-Evans Biosciences, Hangzhou, China). After 48 h, the MenSCs grew by adhering to the wall and nonadherent hematopoietic cells were discarded. The MenSCs used in experiments were at 3rd to 8th passage. Human liver cancer cell lines HepG2 and HuH-7 and human fibroblast MRC-5 were obtained from the National Infrastructure of Cell Line Resources (China). HCC cells and MRC-5 cells were cultured in MEM GlutaMAX (Gibco, USA) with 10% FBS under a 5% CO_2_ atmosphere.

Transwell chamber system with a 0.4-μm pore size was utilized to construct the cell coculture model. To identify the specific impact of MenSCs on HCC cells, we considered HCC cells cocultured with MRC-5 cells or with medium only served as control groups. HCC cells were seeded into the lower chamber, and MenSCs or MRC-5 cells were seeded into the upper chamber in the same medium. HCC cells were collected after coculturing with MenSCs or MRC-5 cells for 24, 48, or 72 h, respectively.

### Methylated DNA immune-precipitation sequencing (MeDIP-Seq) and hydroxymethylated DNA immunoprecipitation sequencing (hMeDIP-Seq)

Purified genomic DNA was sonicated to obtain fragments with a size range of 200–800 bp for hMeDIP-seq or 200–500 bp for MeDIP-seq. Following end repair of the DNA samples with the addition of deoxyadenosine and adaptor ligation based on the Illumina paired-end protocol, the DNA fragments were immunoprecipitated with anti-5-hydroxymethylcytosine or anti-5-methylcytosine antibody. Then, the precipitated fragments were amplified by PCR, and 300–900-bp DNA fragments were selected by using AMPure XP beads. The completed libraries were denatured with 0.1 M NaOH to generate single-stranded DNA molecules, captured on an Illumina flow cell, and amplified in situ. The libraries were then sequenced on the Illumina HiSeq 4000 following the HiSeq 3000/4000 SBS Kit (300 cycles) protocol. After base calling, low-quality reads were passed, and the clean reads were aligned to the UCSC human reference genome hg19 using HISAT2 software (V2.1.0).

### RNA-Seq

Total RNA was isolated from cancer cells by using the AllPrep® DNA/RNA Mini Kit (Qiagen, Germany) according to the manufacturer’s instructions. After assessment of the quantity, quality, and integrity of RNA, the mRNA was enriched with Oligo (dT) beads and rRNA was removed using the Ribo-Zero™ Magnetic Gold Kit (Epicentre, USA). The mRNA fragment library was prepared using the KAPA Stranded RNA-Seq Library Prep Kit (Illumina, USA) according to the manufacturer’s instructions. After mRNA amplification using qPCR, library quality was assessed using the Agilent 2100 Bioanalyzer. The mRNA fragment library was sequenced on the Illumina HiSeq 4000.

### RT-PCR

A total of 100–500 ng RNA was used to synthesize cDNA with the PrimeScript™ RT Reagent Kit (Takara, Japan). The mRNA expression levels of TET1/2/3 and DNMT1/3A/3B were analyzed using SYBR® Premix Ex Taq™ (Takara, Japan) by real-time PCR. The β-actin gene was used as an internal reference. The sequences of primers for RT-PCR are shown in (Additional file 1: Table S1). Relative changes in gene expression were calculated according to the 2^-△△CT^ method.

### Immunohistochemical analysis

Immunohistochemical analysis was performed as described in our previous study. Briefly, all tumor xenograft sections were deparaffinized with xylene and rehydrated through an alcohol gradient, and then, endogenous peroxidase was inactivated by 0.5% H_2_O_2_ for 10 min. Subsequently, the sections were blocked with 5% normal goat serum for 1 h and washed three times with PBS, followed by incubation at 4 °C overnight with one of the following primary antibodies: anti-5-hmC (1:100, Active Motif), anti-5mC (1:100, Active Motif), anti-TET1 (1:300, Abcam), anti-TET2 (1:300, Abcam), anti-TET3 (1:300, Abcam), anti-DNMT1 (1:100, Abcam), anti-DNMT3A (1:500, Abcam), anti-DNMT3B (1:100, Abcam), anti-Ki 67 (1:200, Abcam), and anti-luciferase (1:1000, Abcam). The sections were incubated with horseradish peroxidase (HRP)-conjugated secondary antibody at room temperature for 1 h and developed with a DAB kit.

### Immunofluorescence staining

HCC cells were fixed with 4% paraformaldehyde for 15 min and permeabilized with 0.1 Triton X-100 in PBS for 20 min. To denature the DNA, the cells were treated with 4 M HCl for 15 min at room temperature and neutralized with 100 mM Tris-HCl (pH 8.5) for 10 min. After washing with PBS, the cells were blocked with 5% BSA for 1 h. Then, the cells were incubated with primary antibodies overnight at 4 °C. After washing with PBS, the cells were incubated with secondary antibody (Alexa Fluor 488 goat anti-rabbit IgG Abcam; Alexa Fluor 633 goat anti-mouse IgG, Thermo Fisher) and then stained with DAPI. The plates were scanned, and images were obtained with an Olympus IX83 microscope.

### Western blotting

HCC cells were lysed in ice-cold RIPA lysis buffer with protease inhibitor and phosphatase inhibitor. Protein concentration was determined by using the BCA method. Thirty micrograms of total protein was separated by SDS-PAGE and transferred onto PVDF membranes (Millipore, USA). The membranes were blocked in 0.5% BSA for 1 h at room temperature and incubated overnight with antibodies to PTEN, FOXO3, PI3K, AKT, p-AKT, ERK1/2, p-ERK1/2, C-myc, active caspase3, Bcl2, Bax, E-cadherin, TIMP1, MMP2, snail/slug, twist, and GAPDH (Abcam, USA). Then, the membranes were incubated with the corresponding HRP-labeled secondary antibodies for 1 h at room temperature. Chemiluminescent signals were captured by the Imager (Tiangen, China).

### Animal work

All animal experiments were approved by the Ethics Committee of the First Affiliated Hospital, Zhejiang University. Four-week Balb/c nude mice were obtained from Slac Laboratory Animal Corporation (Shanghai, China). To generate the xenograft model, 2 × 10^6^ HepG 2 cells in 100-μl serum-free medium were implanted subcutaneously into 10 mice at the right foreleg. When the diameter of the tumor under the skin reached 5 mm (about 21 days), 10 mice were randomly divided into two groups (*n* = 5 per group). The MenSC therapy group was treated with tail vein administration of 5 × 10^5^ MenSCs labeled with luciferase in 400-μl serum-free medium every 6 days for three successive times, and the control group received 400-μl serum-free medium only. After first tail vein administration, the tumor volume of each mouse was calculated as (length × width × width)/2 every 3 days until the mice were sacrificed. To evaluate the tumor-tropism of MenSCs, bioluminescence optical imaging was obtained 3 days after the last MenSC treatment. Mice were sacrificed after the bioluminescence optical imaging (about 36 days after HepG 2 implantation), and the xenograft tumor was collected for immunohistochemical analysis.

### Flow cytometry analysis for surface markers of MenSCs and apoptosis of HCC cells

After collection and washing twice with stain buffer (BD Biosciences, CA, USA), MenSCs were incubated with antibodies of surface markers including CD29, CD34, CD45, CD73, CD90, CD105, CD117, and HLA-DR (Becton Dickinson, NJ, USA) for 20 min. The stained cells were washed twice with stain buffer and resuspended in 500 μl of stain buffer to be analyzed by FC500 flow cytometer (Beckman Coulter, CA, USA).

HCC cells were seeded in six-well plates and cocultured with MenSCs, MRC-5, and medium only for 72 h, respectively. Cell apoptosis was detected using Annexin V/PI detection kit (BD Biosciences, CA, USA) by FC500 flow cytometer. Data were analyzed using Flowjo software (Tree Star, OR, USA).

### Cell viability assay

HCC cells and MenSCs were seeded into the 24-well plates and upper transwell chambers, respectively. Cell viability was assessed by Cell Counting Kit-8 (Dojindo) according to the manufacturer’s protocol.

### Clonogenic assay

HCC cells were seeded in six-well plates at a density of 1000 cells per well and cocultured with MenSCs, MRC-5, and medium only using transwell coculture system for 7 days. After incubation, HCC cells were fixed with 4% paraformaldehyde and stained with 0.1% (*w*/*v*) crystal violet. Megascopic cell colonies were calculated under an Olympus IX83 microscope, and each measurement was performed in triplicate.

### 5-hmC ELISA

For measuring the global DNA hydroxymethylation levels, we used MethylFlash™ Global DNA hydroxymethylation (5-hmC) ELISA Easy Kit (Epigentek) to detect the 5-hmC level in HCC cells after MenSC treatment. ELISA was performed according to the manufacturer’s protocol.

### Wound healing assay

HCC cells were seeded in IBIDI wound healing system in 12-well plates. After cells reached a confluence of more than 90%, removing the IBIDI stands to form a straight line without cells in the middle of the wells. Then, HCC cells were cocultured with MenSCs, MRC-5, and medium only, respectively. Photographs were taken from 0 to 48 h on an optical microscope.

### Transwell invasion assay

The upper chambers with 8-μm pore size were precoated with 1 mg/ml Matrigel (BD Biosciences, USA) for 1 h. 2 × 10^4^ of HCC cells in 100-μl medium were added into upper chambers, and 2 × 10^4^ of MenSCs and MRC-5 cells were seeded in the lower chamber beforehand. After 48-h incubation, HCC cells were fixed with 4% paraformaldehyde and stained with 0.1% crystal violet. Stained cells were observed and counted under a microscope.

### Statistical analysis

All data analysis was conducted using SPSS 18.0 statistical software and GraphPad Prism 6 software. All values were expressed as the means ± SD. Student’s *t* test was used for determining significant differences between groups. Two-way ANOVA was used to analyze CCK8 assay. The difference of two groups from five time points in CCK8 assay was performed by Sidak’s multiple comparisons test. Functional enrichment analysis was performed by pathway analysis using the DAVID web server (http://david.abcc.ncifcrf.gov/). Genes with DMRs and DHMRs in enhancers were mapped to their respective human orthologs, and the lists were submitted to DAVID for enrichment analysis to determine significant KFGG-pathway categories. For time-to-event analyses, survival estimates were calculated by the Kaplan-Meier analysis, and groups were compared with the log-rank test. *P* < 0.05 was considered statistically significant.

## Results

### MenSCs had an anticancer effect associated with dynamic DNA methylation alteration

**Fig. 1:**
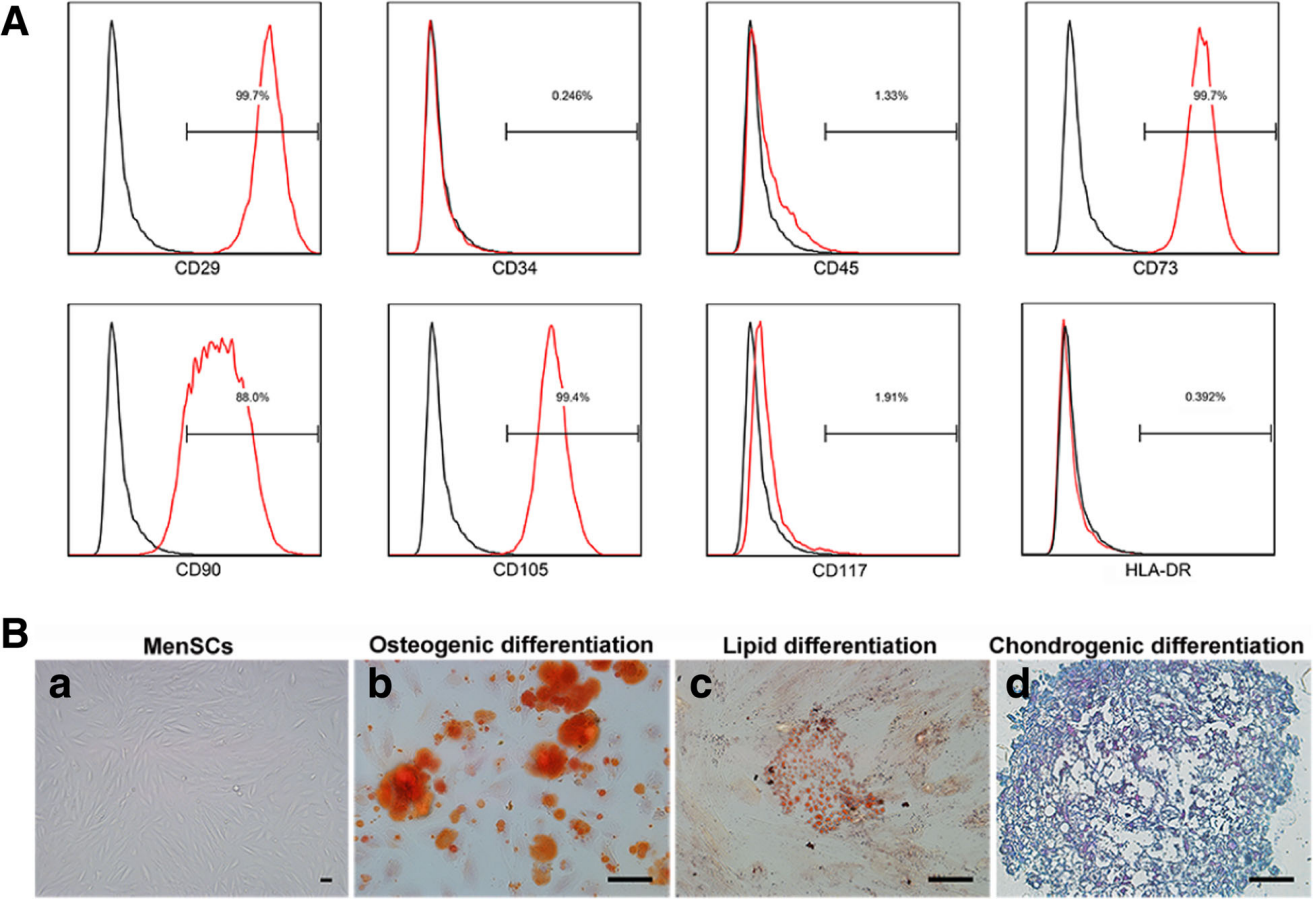
Characterization of MenSCs. **a** Flow cytometry analysis showed surface markers of MenSCs. **b** Representative images showed morphology and tri-lineage differentiation potential of MenSCs. Scale bar 50 μm

Flow cytometry was performed to identify the phenotype of MenSCs, and the results showed that MenSCs were positive for CD29, CD73, CD90, and CD105 and negative for CD34, CD45, CD117, and human leukocyte antigen DR (HLA-DR) (Fig. 1a). Moreover, MenSCs were able to differentiate into adipocytes, chondroblasts, and osteoblasts (Fig. 1b). Thus, MenSCs had low immunogenicity and multiple differentiation potential and were suitable for transplantation into animals or humans.

**Fig. 2:**
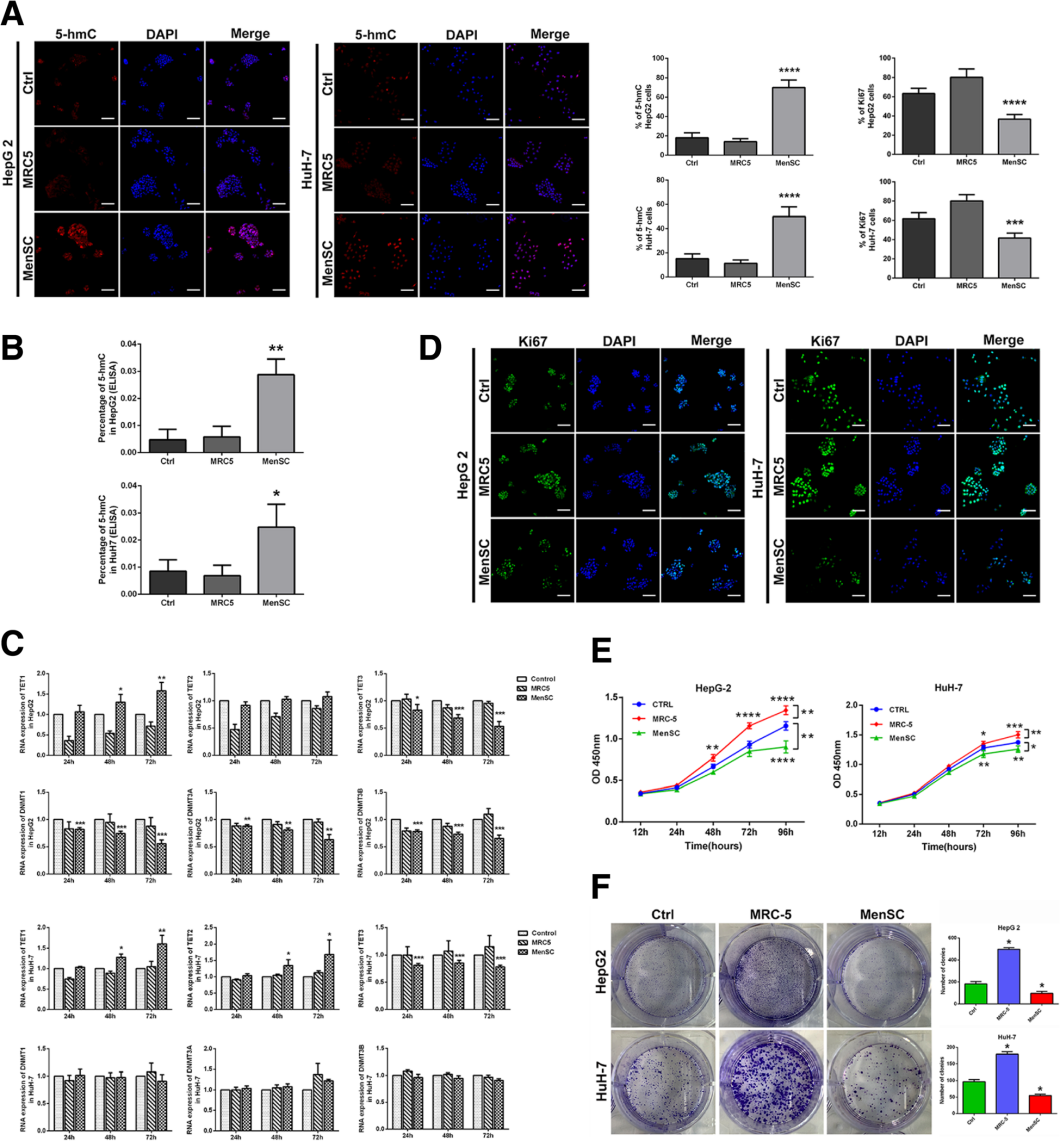
MenSCs had an anticancer effect associated with dynamic epigenetic alterations in vitro. **a** 5-hmC level in HCC cells was measured using the IF method. **b** Percentage of 5-hmC in genome was detected using ELISA. **c** Expression of DNA methylation and demethylation enzymes in HCC cells was altered in a time-dependent manner during MenSC coculture. **d** Ki67 was measured using the IF method to show the proliferation state of HCC cells. **e** CCK8 assay was performed to determine the cell viability of HCC cells after 96 h of MenSC coculture. **f** Clonogenic assay was evaluated to determine the potential of clonal formation in HCC cells with MenSC coculture. Scale bar 100 μm. *P < 0.05, **P < 0.01, ***P < 0.001, ****P < 0.0001

To investigate whether MenSCs play a role in the epigenetic regulation of HCC cells, we measured the 5-hmC level of HCC cells after MenSC coculture for 72 h using immunofluorescence (IF) assay and ELISA assay. IF assay showed that 5-hmC level in HCC cells was significantly increased after MenSC coculture (Fig. 2a). ELISA assay further showed the 5-hmC level of HCC with MenSC coculture was significantly increased than that of the control group (HepG2 0.0288 ± 0.0033 vs 0.0047 ± 0.0022, *P* = 0.0038; HuH-7 0.0248 ± 0.0049 vs 0.0085 ± 0.0024, *P* = 0.0415, Fig. 2b). Then, we detected DNA methylation and demethylation enzymes, including TETs and DNMTs in HCC cells, through a real-time PCR assay. Results showed that TET1 expression in HepG2 was gradually enhanced in a time-dependent manner from 24 to 72 h during MenSC coculture. However, TET3 expression and that of the DNMTs, especially DNMT1, gradually decreased (Fig. 2c). Additionally, TET1 and TET2 expression was clearly enhanced, while DNMTs did not appear to decrease evidently in HuH7 under MenSC coculture (Fig. 2c). These results suggested that MenSCs dynamically regulate DNA methylation in diverse HCC cells via different methylation enzymes. Next, we measured the biological effect of MenSCs on HCC growth and evaluated whether the epigenetic alterations mediated by MenSCs were associated with cell growth. IF assay showed that Ki67 levels in HepG2 and HuH-7 were obviously decreased after MenSCs coculture for 72 h (Fig. 2d). Furthermore, CCK8 assays showed that, compared with the controls, MenSCs significantly suppressed the proliferation of HCC cells (Fig. 2e). However, MRC-5 clearly promoted the proliferation of HCC cells, indicating that fibroblast cells could fuel cell growth in the tumor microenvironment. Additionally, MenSCs inhibited the clonogenic potential of HepG2 and HuH-7 (Fig. 2f). Compared with control groups, the clone number of the MenSC group was significantly decreased. These results indicated that MenSCs had a suppressive impact on the growth of HCC cells in vitro. Moreover, the suppressive effect of MenSCs on the HCC growth was associated with epigenetic alterations.

**Fig. 3:**
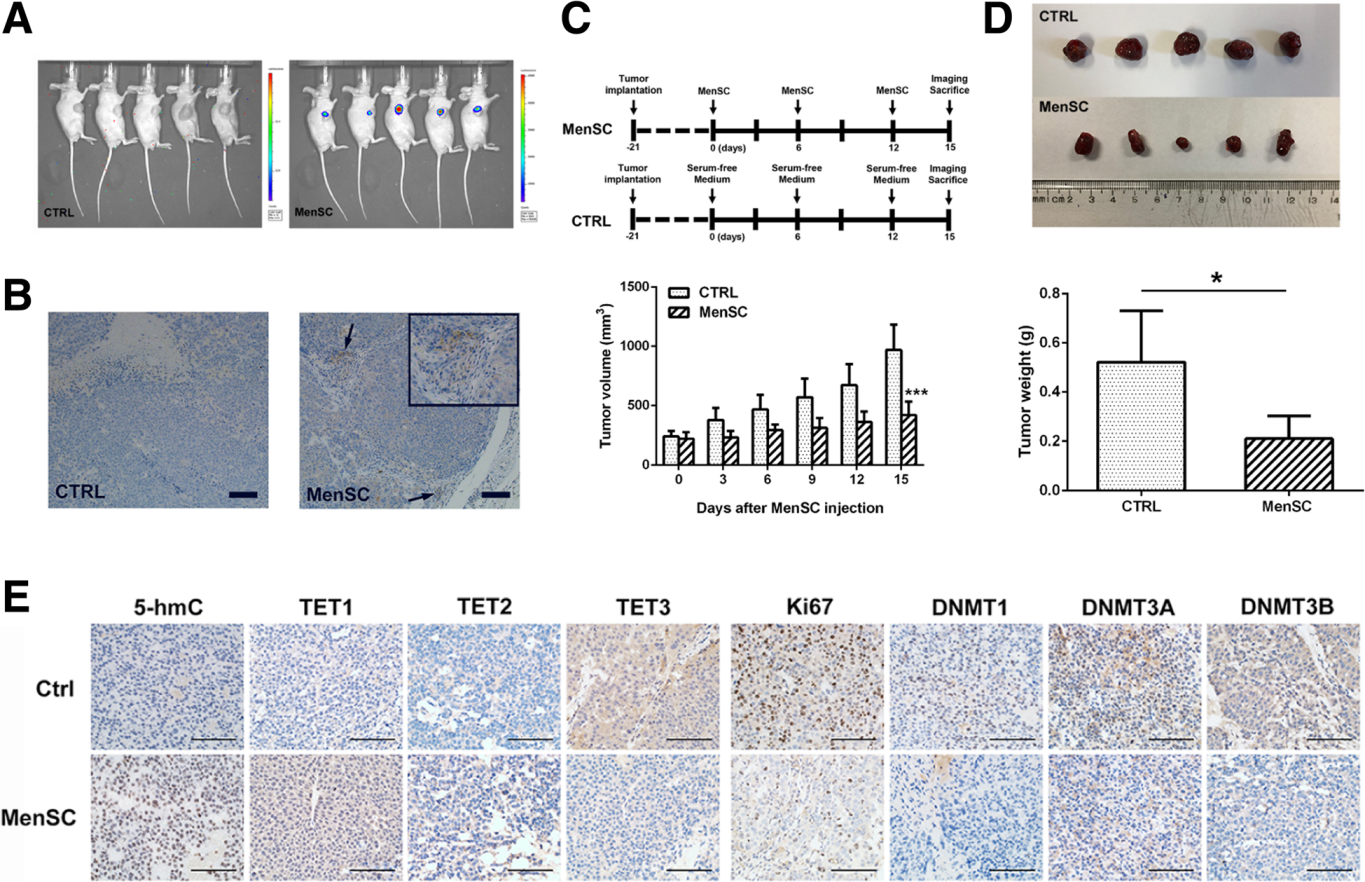
Epigenetic regulations mediated by MenSCs in vivo. **a** Schematic diagram of the treatment with MenSCs that carried luciferase and serum-free medium in nude mice bearing xenograft HCC cells. **b** IHC assay displayed the distribution of MenSCs in HCC tissues. **c** Comparison of tumor volume between the MenSC group and control group during the period of MenSC implantation. **d** Comparison of tumor weight between two groups after three times of MenSC implantation. **e** IHC assay showed alterations in 5-hmC, Ki67, TETs, and DNMTs in HCC tissues with MenSC therapy. Scale bar 100 μm. *P < 0.05, ***P < 0.001

Furthermore, we wanted to validate whether MenSCs play a role in epigenetic regulation of HCC in vivo. As shown in Fig. 3a and b, MenSCs that carried luciferase could specifically target the tumor site and we also used the antibody of anti-luciferase to track the distribution of MenSCs in HCC tissues. Tumor volume and weight of mice injected with MenSCs were significantly lower than those of the control group after three times of tail vein injection (Fig. 3c, d). IHC assay showed the 5-hmC level in HCC with MenSC injection was significantly higher than that in the control group (Fig. 3e). Moreover, the expression of TET1 and TET2 was clearly increased, and the expression of TET3, DNMT1 DNMT3A, DNMT3B, and Ki67 were sharply reduced in HCC with MenSC injection. Collectively, these results demonstrated that MenSCs could suppress the growth of HCC and modify epigenetic regulation in vivo.

### MenSCs influenced global patterns of hydroxymethylome and methylome in HCC

**Fig. 4:**
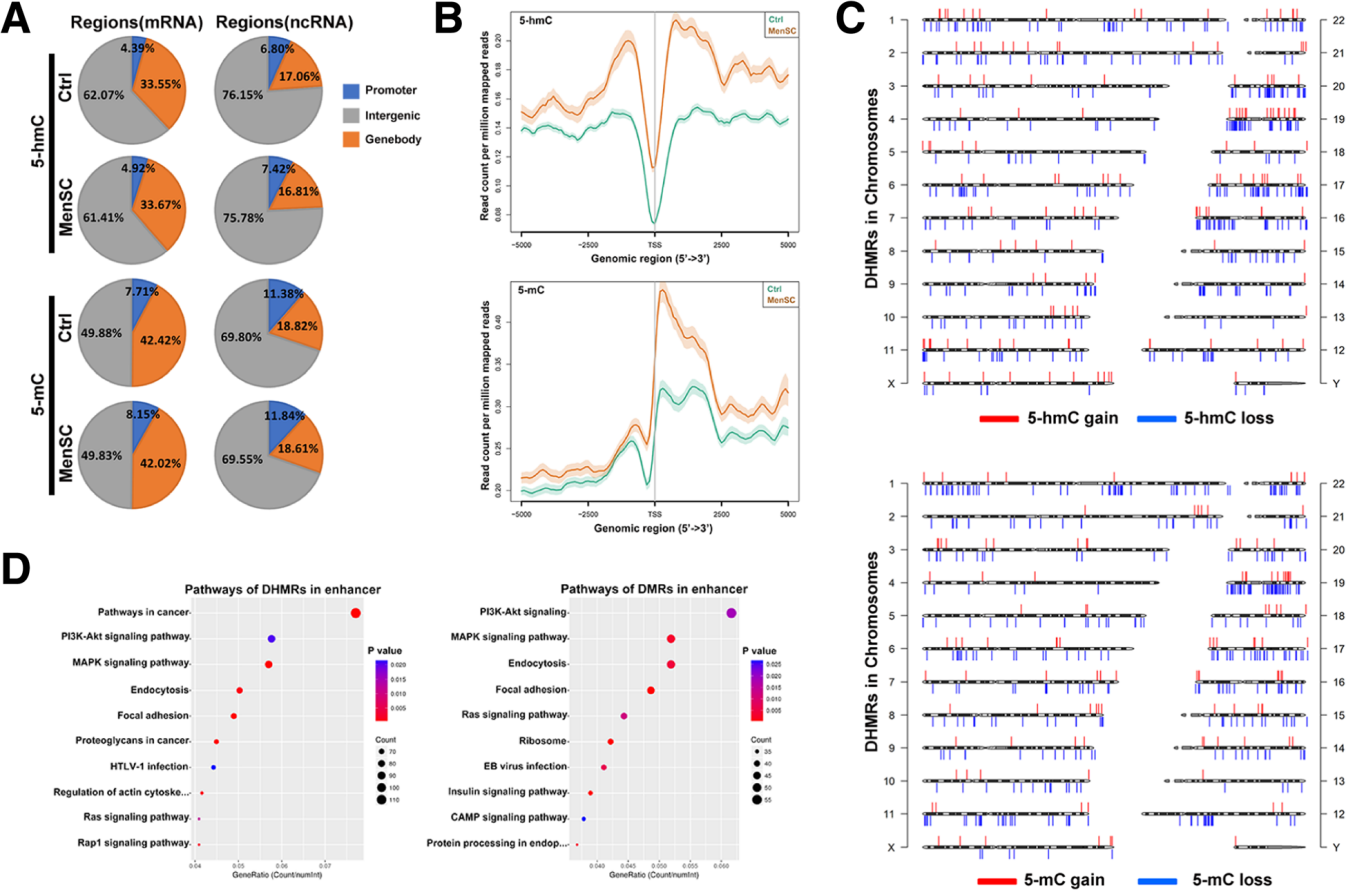
Landscapes of 5-hmC and 5-mC in HCC cells after MenSC therapy. **a** 5-hmC and 5-mC distribution in different genomic regions including promoter, gene body, and intergenic regions. **b** Distribution of 5-hmC and 5-mC in the 5-kb upstream and downstream flanking regions of the TSS in HCC cells with or without MenSC treatment. **c** Distribution of DHMRs and DMRs in all chromosomes in HCC cells after MenSC therapy. **d** Top pathways of DHMRs and DMRs in enhancer regions. The dot plot shows the gene ratio values of the top ten most significantly enriched pathways; the dot size indicates the number of corresponding genes, and the color shows the *P* value

Next, we used the hMeDIP-seq and MeDIP-seq technologies to meticulously analyze whole-genome changes in methylome and hydroxymethylome of HepG2 after 72 h of MenSC coculture. We mapped the distribution of all hydroxymethylated and methylated signals in HCC cells (Fig. 4a). In intergenic regions, the proportion of 5-hmC signal was more abundant than that of the 5-mC. However, in promoter and genebody regions, 5-mC level was more affluent than 5-hmC level. Moreover, the alterations of 5-hmC abundance tended to occur in promoter and intergenic regions after MenSC coculture, while 5-mC changes mainly happened in promoter regions (Fig. 4a). By graphing 5-hmC and 5-mC distribution across gene regions, we showed that 5-hmC and 5-mC formed a deep dip around transcription start sites (TSSs) (Fig. 4b). Moreover, 5-mC formed a large peak to the right of TSSs. Both 5-mC and 5-hmC exhibited several small peaks along gene bodies toward the 3′ gene terminus. Additionally, the abundance and distribution of 5-hmC and 5-mC in HCC cells exhibited significant alterations after MenSC coculture. These results suggested that changes of 5-hmC and 5-mC in different regions mediated by MenSCs may play different roles in transcription regulation.

Next, we mapped the distribution of differentially hydroxymethylated regions (DHMRs) and differentially methylated regions (DMRs) in all chromosomes. We found that alterations in 5-hmC and 5-mC occurred throughout the genome after MenSC treatment. Moreover, DHMRs and DMRs generally did not overlap in chromosomes (Fig. 4c). Furthermore, we aimed to explore which pathways were significantly differentially regulated in conjunction with MenSC-mediated epigenetic alterations. Interestingly, we found genes which have DHMRs or DMRs in enhancers were primarily associated with MAPK pathway, FOXO pathway, PI3K/Akt pathway, focal adhesion, endocytosis, and apoptosis (Fig. 4d, Additional file 1: Table S2 and S3). These results suggested that MenSCs might secrete various components to induce the activation of endocytosis of HCC cells and influence the microenvironment to regulate multiple biological functions including apoptosis and metastasis via epigenetic alterations.

### MenSCs promote HCC cell apoptosis through the PI3K/AKT/FOXO pathway via regulating enhancer 5-hmC and 5-mC levels

**Fig. 5:**
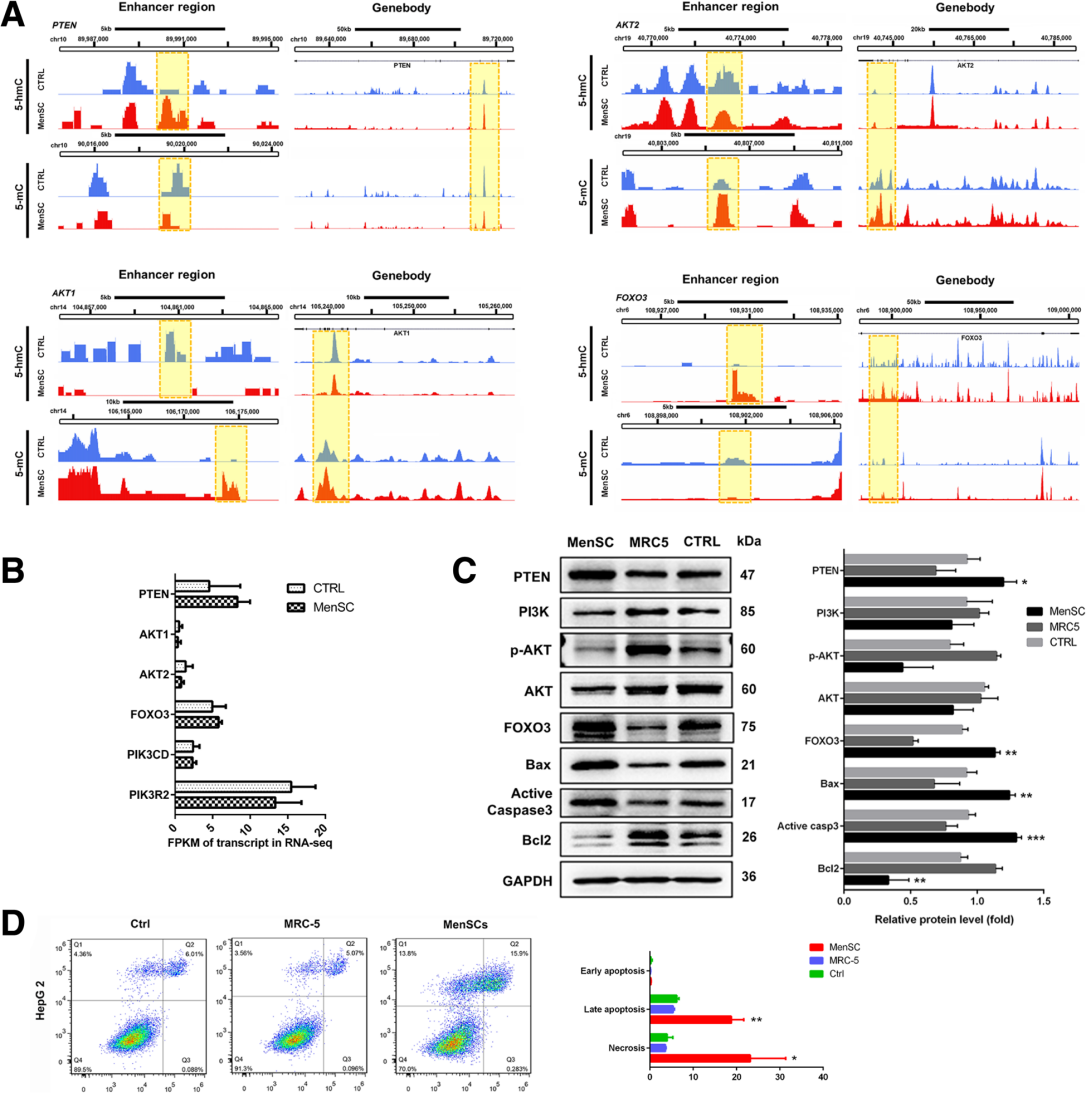
MenSCs suppress PI3K/AKT pathway via regulating 5-hmC and 5-mC abundance at regulatory regions. **a** 5-hmC and 5-mC peaks in enhancers or around TSS regions in PTEN, AKT1, AKT2, and FOXO3 were significantly changed after MenSC treatment. Yellow boxes indicate the location of DHMRs and DMRs. **b** FPKM of transcript for key genes from RNA-Seq data. **c** Western blot assay indicates that protein expression of pivotal genes in the PI3K/AKT pathway after MenSCs coculture and that downstream apoptosis was activated. **d** Cell apoptosis and necrosis of HCC cells after MenSC treatment were clearly increased by flow cytometry with FITC-Annexin V/PI double staining. *P < 0.05, **P < 0.01, ***P < 0.001

Given that pathway analysis presented hydroxymethylated and methylated alterations were closely associated with PI3K/AKT pathway, we next verified which genes were regulated by epigenetic alterations. PTEN is the key negative regulatory factor of the PI3K/AKT pathway, and we found that the 5-hmC level in enhancer region of PTEN (chr10:89989801-89990380) was significantly increased (*P* = 6.06E−07), while the 5-mC level in the enhancer (chr10:90019261-90019640) was decreased following MenSC coculture (*P* = 2.07E−09; Fig. 5a). However, the 5-hmC and 5-mC abundances in enhancer regions of Akt1 (*P* = 3.31E−5) and Akt2 (*P* = 5.21E−5) were apparently altered in response to MenSC treatment. Moreover, MenSCs induced a reduction in 5-hmC in the enhancer region of PIK3CD (*P* = 2.18E−05) and an increase in 5-mC in the enhancer region of PIK3R2 (*P* = 1.09E−05) to suppress the PI3K-mediated activation of Akt (Additional file 2: Figure S1). In addition, the FOXO pathway is one of the downstream targets of Akt and involves in the cell apoptosis. The 5-hmC abundance in the enhancer of FOXO3 was also upregulated (chr6:108929921-108930200; *P* = 2.53E−6; Fig. 5a). Additionally, promoter regions of apoptosis-related genes, including TNFRSF1A and Caspase7, showed significant increases in 5-hmC abundance (Additional file 2: Figure S1). To explore whether alterations in 5-hmC and 5-mC in these regions influenced gene expression, we determined RNA and protein expression in HCC cells. Results showed that MenSCs could significantly upregulate PTEN and FOXO3 expression compared with control groups (Fig. 5b, c). PI3K, Akt, and phosphorylated Akt proteins were sharply decreased under coculture with MenSCs. Furthermore, downstream apoptotic proteins Bax and cleaved caspase3 were evidently enhanced, while Bcl-2 was reduced (Fig. 5c). Moreover, flow cytometry assay confirmed that HepG 2 cocultured with MenSCs displayed significantly higher proportions of late apoptotic cells (18.80 ± 1.65 vs 6.19 ± 0.30, *P* = 0.0017) and necrotic cells (23.17 ± 4.74 vs 3.98 ± 0.78, *P* = 0.0162, Fig. 5d). These results indicated that MenSCs promotes apoptosis of HCC through suppression of PI3K/AKT pathway via regulating 5-hmC and 5-mC at enhancers.

### MenSCs upregulate E-cadherin via repressing ERK-MYC-mediated EMT in HCC

**Fig. 6:**
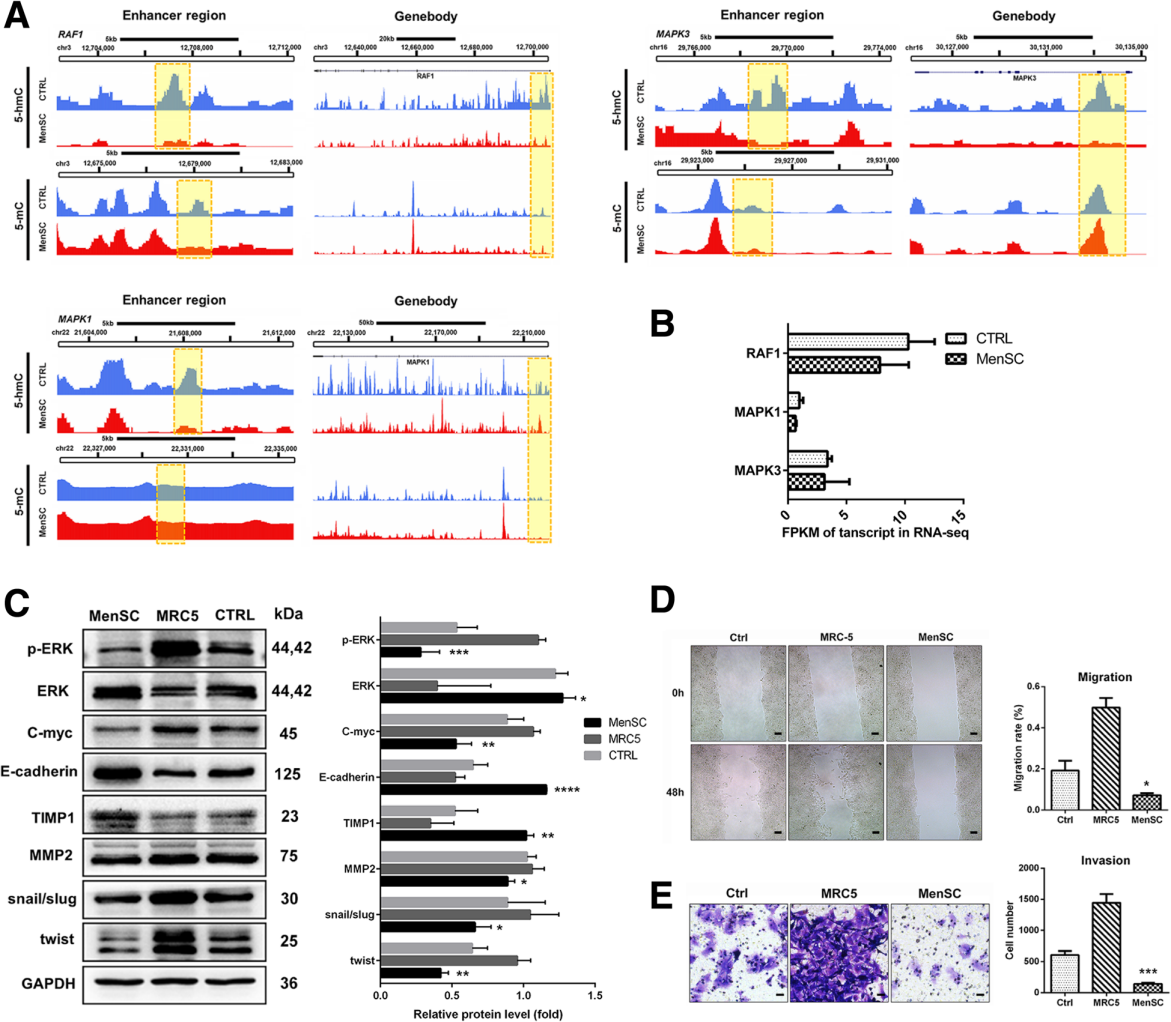
MenSCs inhibit EMT of HCC through suppressing MAPK pathway at 5-hmC and 5-mC regulatory regions. **a** 5-hmC levels in the enhancer and promoter regions of RAF1, MAPK1, and MAPK3 were clearly downregulated. The 5-mC level of an enhancer in MAPK1 was increased, while those in RAF1 and MAPK3 showed no significant increase. **b** FPKM of transcript for MAPK pathway genes correspond to the epigenetic alterations. **c** Protein expression of key genes in the MAPK pathway after MenSC treatment. Downstream EMT was suppressed by MenSC coculture. **d**, **e** Cell migration and invasion of HCC cells measured by wound healing assay and transwell assay after MenSC coculture. Scale bar 50 μm. *P < 0.05, **P < 0.01, ***P < 0.001, ****P < 0.0001

The RAF1-ERK pathway plays a vital role in HCC carcinogenesis, progression, and chemotherapy resistance. Hence, we focused on whether MenSCs had a suppressive impact on the MAPK pathway through epigenetic alterations. The results showed that MenSCs suppressed the MAPK pathway not only via changing the 5-hmC and 5-mC levels in the enhancer regions but also through regulating the promoter regions. The 5-hmC level of RAF1 in both the enhancer (chr3:12706241-12706880; *P* = 1.97E−7) and promoter (chr3:12706241-12706880; *P* = 1.97E−7) was significantly decreased under MenSC coculture (Fig. 6a). In addition, 5-hmC levels of MAPK1 (chr22:21607661-21608260; *P* = 1.10E−7) and MAPK3 (chr16:29768301-29768540; *P* = 1.22E−5) in enhancer regions were clearly decreased. Moreover, MenSCs also reduced the 5-hmC level in the promoter region of MAPK3 (chr16:30132781-30133160; *P* = 6.55E−6). The mRNA expression of MAPK1, MAPK3, and RAF1 correlated with the epigenetic changes (Fig. 6b). Additionally, phosphorylated ERK1/2 in the MenSC group was nearly absent compared with that in the control group (Fig. 6c). C-myc, the downstream target of ERK1/2, was significantly reduced after MenSC coculture. These results indicated that phosphorylation of ERK1/2 was a prerequisite for the activation of c-myc in HCC. Such changes in DNA epigenetic modification probably had a cascading influence on gene expression, including posttranscriptional regulation and posttranslational modification. Furthermore, we observed that MenSCs could upregulate E-cadherin and TIMP1 and downregulate the MMP2, twist, and snail-slug proteins (Fig. 6c). To verify whether MenSCs could inhibit the metastasis and invasion of HCC in vitro, we conducted a wound healing assay and invasion assay. Compared with control groups, the metastasis and invasion ability of HCC cells was significantly inhibited by MenSCs (Fig. 6d, e). Hence, we speculated that MenSCs hampered the metastasis of HCC cells via suppression of ERK-MYC-EMT axis through epigenetic regulation.

### MenSCs downregulated drug resistance genes and activated tumor suppressors via epigenetic mechanisms

**Fig. 7:**
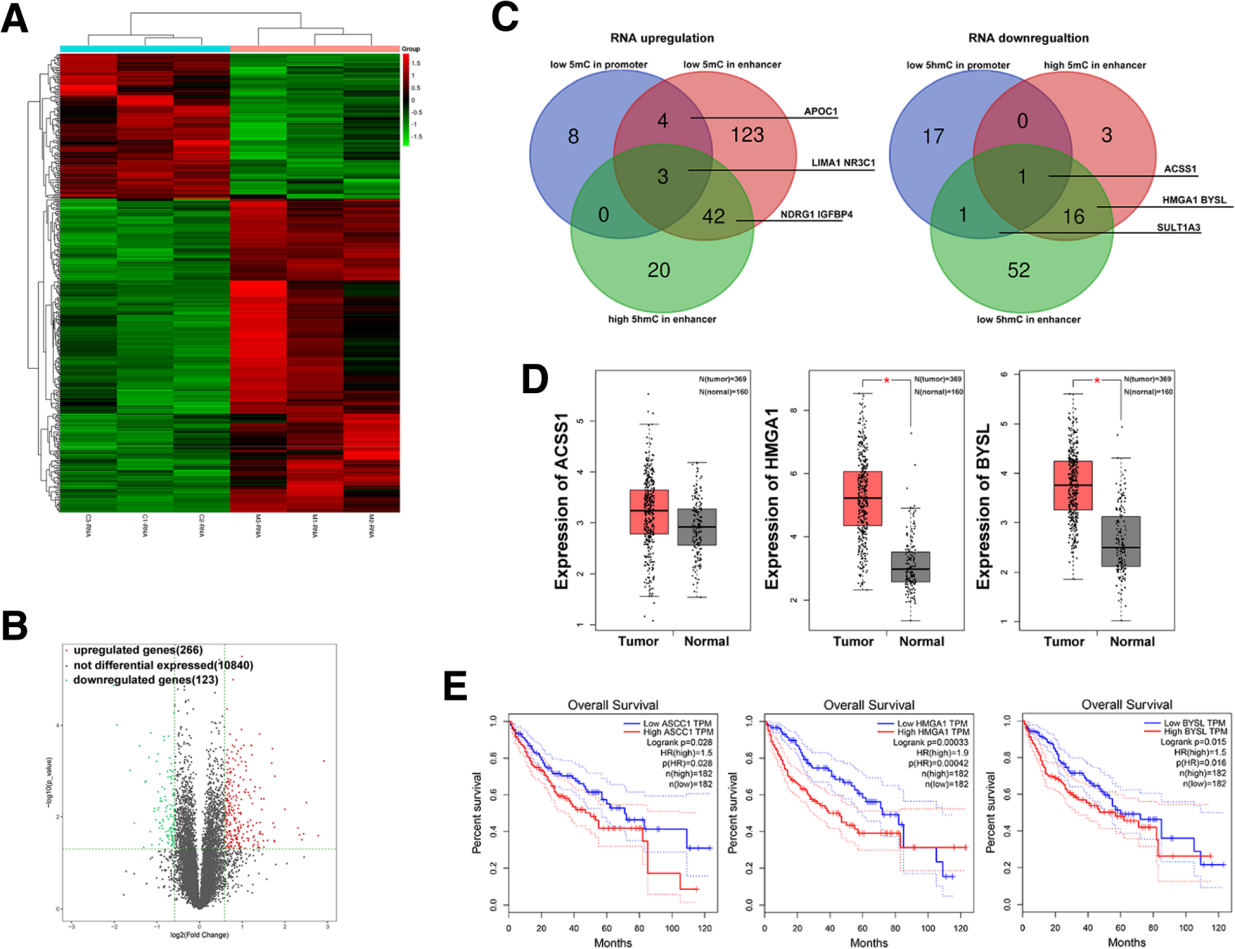
MenSCs induce differential expressed genes associated with 5-hmC and 5-mC changes. **a** Hierarchical clustering of RNA-seq results across 6 HCC cell samples. **b** Volcano plot analysis showed differentially expressed genes between HCC cells with or without MenSCs coculture. **c** Significantly changed genes among three groups from RNA-seq, MeDIP-seq, and hMeDIP-seq data of HCC cells. **d** Expression level of ACSS1, HMGA1, and BYSL in HCC tissues and normal tissues from TCGA database. **e** Expression of ACSS1, HMGA1, and BYSL were associated with overall survival of HCC patients by Kaplan-Meier from TCGA database

Next, we combined RNA-seq, MeDIP-seq, and hMeDIP-seq to identify differentially expressed mRNAs in HCC after MenSC therapy. We observed a clear separation between the MenSC group and the control in the hierarchical clustering analysis of mRNA (Fig. 7a). A total of 266 genes were upregulated, and 123 genes were downregulated in HCC cells with MenSC treatment (Fig. 7b). Based on the integrated analysis, we identified that 206 upregulated genes (206/266, 77.4%) and 92 downregulated genes (92/123, 74.8%) had alterations of 5-hmC and 5-mC at regulatory regions. Among the downregulated genes, we found several drug resistance genes were inhibited via epigenetic regulation (Table 1). Additionally, potential suppressors including LIMA1, NR3C1, IGFBP4, and NDRG1 were upregulated via epigenetic mechanisms (Fig. 7c). Furthermore, ACSS1, HMGA1, and BYSL which closely correlated with tumorigenesis and overall survival were significantly downregulated via 5-hmC and 5-mC alterations at enhancer and promoter (Fig. 7d, e). Hence, we speculated these genes might be potential targets for gene-modified MSC therapy. In addition, MenSCs combined with chemotherapy might be a strategy to alleviate the drug resistance.

**Table 1:** Drug resistance-related genes differentially expressed after MenSC coculture via epigenetic alterations

Genes	Expression after MenSC therapy	Epigenetic alteration after MenSC therapy	Expression in literature	Drug resistance
ID4	Downregulated	5mC upregulation in the promoter 5-hmC downregulation in the enhancer	Upregulated	Glioma: 1,3-bis(2-chloroethyl)-1-nitrosourea
SQSTM1	Downregulated	5-hmC downregulation in the promoter	Upregulated	Ovarian cancer: cisplatin Gastric cancer: cisplatin
HMGA1	Downregulated	5mC upregulation in the enhancer 5-hmC downregulation in the enhancer	Upregulated	Lung cancer: tyrosine kinase inhibitor Ovarian cancer: paclitaxel, doxorubicin Sarcoma: trabectedin Cholangiocarcinoma: gemcitabine
GATA2	Downregulated	5-mC upregulation in the enhancer 5-hmC downregulation in the enhancer	Upregulated	Prostate cancer: docetaxel, cabazitaxel
PSAT1	Downregulated	5-hmC downregulation in the enhancer	Upregulated	Colorectal cancer: oxaliplatin Breast cancer: tamoxifen
FASN	Downregulated	5-hmC downregulation in the enhancer	Upregulated	Ovarian cancer: cisplatin Lung cancer: tyrosine kinase inhibitor Gastrointestinal stromal tumor: imatinib Triple-negative breast cancer: cetuximab

## Discussion

The tumor microenvironment plays important roles in tumorigenesis, progression, and drug resistance. Epigenetic mechanisms may function as an interface between environmental factors and the genome, and thus, deregulation of the epigenome by environmental stressors is likely to disrupt a variety of cellular processes [28]. Accumulating evidence indicates that cancer cells not only reshape their epigenomes in response to an altered milieu but also reprogram the tumor microenvironment toward an immunosuppressive state [29]. MSCs are one of the major types of surrounding cell that constitute the tumor microenvironment. Previous studies have confirmed that extraneous MSCs can travel to tumor sites and exert an anticancer effect by altering the microenvironment through multiple paracrine signaling components [30, 31]. However, no reports have indicated whether MSCs can influence the epigenomes of cancer cells via reshaping the microenvironment. Hence, we here presented evidence that MenSCs exert a crucial influence on HCC epigenetics and have an anticancer effect by epigenetically suppressing oncogenic pathways.

In our study, MenSCs could revive the 5-hmC and TET1 expression of HCC cells in a time-dependent manner. TET1, which has been described as a novel suppressor, was depleted, along with 5-hmC reduction, in multiple solid tumors including hepatocarcinoma [32]. Although the mechanism of TET1 reduction in tumorigenesis remains incompletely understood, multiple lines of evidence indicate that TET1 dysregulation plays a major role in the disturbance of DNA demethylation found in solid cancers. TET2 mutations frequently occur in myeloid malignancies and are accompanied by a decline in 5-hmC and poor prognosis [33]. The mechanism of TET2 inactivation in solid cancer remains elusive, given that TET2 rarely mutates in solid cancer. However, Sajadian et al. [34] found that vitamin C and 5-azacytidine induce active demethylation, arresting the cell cycle in HCC cells, via TET2. In our study, we also observed an apparent change of TET2 expression in HuH-7 after MenSC treatment. Hence, we speculate MenSCs rescued demethylation of HCC cells via TET1 or TET2 in different HCC cells. Moreover, MenSCs combined with chemotherapy such as 5-azacytidine might be a potential therapeutic schedule to activate both TET1- and TET2-mediated demethylation. The role of TET3 in tumorigenesis is still unclear. Most researchers support the viewpoint that TET1 and probably TET2 are downregulated in solid cancers, while TET3 increases its expression to maintain demethylation for DNA repair and cell survival [35]. In our study, TET3 and DNMT1 were repressed under MenSC coculture. These results indicated that upregulation of TET3 for compensation would not be necessary due to the demethylation mediated by TET1 in HCC that was reactivated by MenSCs. Moreover, DNMT downregulation might be beneficial for tumor suppressor revival via maintaining the balance between DNA methylation and demethylation in genomics.

Previous studies confirmed that MSCs inhibit cancer cells via secretion of diverse components into microenvironment. Yulyana et al. [30] considered that MSC-derived media expressed a high level of insulin growth factor binding proteins (IGFBPs) and can sequester free insulin-like growth factors (IGFs) to inhibit HCC cell proliferation. de Araujo Farias et al. [36] found MSC-derived exosomes enhance radiotherapy-induced cell death in tumor and metastatic tumor foci. In our study, we confirmed that MenSCs have a suppressive effect on HCC cells in vitro and vivo through epigenetic regulation via paracrine signaling. Hence, it is indicated that MSCs not only secrete anticancer factors to directly inhibit oncogenic pathways, but also regulate the epigenetic mechanisms of cancer cells to suppress downstream biological functions including apoptosis and metastasis.

Outside of this study, whole-genome hydroxymethylation and methylation profiling have been mainly applied in tumor etiology studies to compare cancer tissues with adjacent tissues. Johnson et al. [37] found that 5-hmC enrichment regions are strongly associated with active transcription in glioblastoma, and 5-hmC was preferentially enriched in glioblastoma-specific enhancers and superenhancers. Additionally, Bhattacharyya et al. [38] revealed that DHMRs were concentrated mostly in regulatory regions and particularly in non-CGIs in pancreatic ductal adenocarcinoma (PDAC). In pancreatic differentiation, DNA hydroxymethylation is positively correlated with enhancer activities and chromatin accessibility [39]. The present study represents the first time that hMeDIP-seq, MeDIP-seq, and RNA-seq have been combined to demonstrate the impact of MenSC therapy on epigenetic regulation in HCC cells. We performed genome-wide mapping of the hydroxymethylome and methylome landscapes of HCC cells after MenSC therapy. Most DHMR and DMR localization occurred in the regulatory regions of the genome, such as the enhancer, superenhancer, and promoter regions. These findings demonstrate that dynamic changes in 5-hmC and 5-mC in enhancer regions represent a crucial epigenetic mechanism for regulating gene expression in cell differentiation and tumorigenesis.

PTEN is a major inhibitor of the PI3K/AKT pathway, which is frequently silenced via mutation or promoter hypermethylation in various cancers. Loss of PTEN or activation of PI3K/AKT is involved in cancer progression and drug resistance [40, 41]. However, inhibition of Akt reverses acquired resistance to sorafenib by switching protective autophagy to autophagic cell death in HCC [42]. Lee et al. [43] found that loss of the tumor suppressor IGFBP4, which constitutes an AKT-EZH2 reciprocal loop, drives H3K27me3-mediated epigenetic reprogramming in hepatic carcinogenesis. In the present study, we showed that MenSCs inhibit the PI3K/AKT pathway through regulating 5-hmC and 5-mC abundance in the enhancer regions of key genes. These results suggested that the redistribution of 5-hmC abundance mediated by MenSCs in HCC cells is associated with transcriptional activity. Sorafenib is indicated as a treatment for advanced HCC. However, the clinical efficacy of sorafenib is severely compromised by the development of drug resistance. Recently, Kim et al. [44] found that RAF/MEK/ERK signaling was activated by sorafenib and caused sorafenib resistance in HCC. Moreover, phospho-ERK might be a biomarker of response to a synthetic lethal drug combination of sorafenib and MEK inhibition in liver cancer [45]. In the present study, MenSCs suppressed the RAF/ERK/MYC pathway via regulation of 5-hmC and 5-mC levels in enhancer and promoter regions. Furthermore, reductions in phosphorylated ERK1/2 and c-myc repressed downstream EMT. Hence, MAPK pathway would be a vital target for the strategy of modified gene MSC. FOXO3 acts downstream of the PI3K/AKT and RAF/ERK pathways to govern multiple cellular processes including the cell cycle, apoptosis, autophagy, and metabolism. Our study demonstrated that MenSCs could induce the expression of FOXO3 and further promote cell apoptosis in HCC. Fitzwalter et al. [46] found that FOXO3 acts as a cell surveillance mechanism to correct autophagy perturbations and confers apoptosis sensitization if this autophagy imbalance is not rectified.

Demethylation chemotherapeutic drugs, including 5-azacytidine and 5-aza-2′-deoxycytidine, cause whole-genome demethylation and randomly activate the expression of silenced genes. However, we found that MenSCs specifically modulate the expression of target genes through altering 5-hmC and 5-mC abundances in different regions. For instance, MenSCs rescue suppressors, IGFBP4 and NDRG1, via upregulation of 5-hmC and downregulation of 5-mC in enhancer regions, while suppressing ACSS1 expression via downregulation of 5-hmC in enhancer and promoter regions and upregulating 5-mC in enhancer regions. BYSL protein is upregulated in HCC and required for nucleologenesis in cancer cell proliferation [47]. Overexpression of HMGA1 is significantly associated with tumor progression and EMT in multiple cancers [48, 49]. Suppression of BYSL and HMGA1 might be potential strategy for HCC therapy.

## Conclusions

MenSCs regulated transcriptional activities of PI3K/AKT and RAF/ERK via alteration of 5-hmC and 5-mC abundance in gene regulatory regions in HCC cells. Deactivation of PI3K/AKT and RAF/ERK signaling attenuated the inhibition of FOXO3 and promoted downstream apoptosis. Moreover, an increase in PTEN further strengthened the suppressive effect on PI3K/AKT. Additionally, inactivation of phosphorylated ERK repressed c-myc-mediated EMT. Taken together, our results provided important epigenetic evidence for clarifying the mechanism of crosstalk between cancer and MSCs in the microenvironment and screening effective epigenetic targets for future therapy strategies based on modified MSCs.

## Additional files

Additional file 1: **Table S1.** The sequences of primers for RT-PCR. Table S2. Top 20 pathways of DHMRs in enhancer regions in HCC cells after MenSC coculture. Table S3. Top 20 pathways of DMRs in enhancer regions in HCC cells after MenSC coculture. (XLSX 76 kb)

Additional file 2: **Figure S1.** Alterations of 5-hmC and 5-mC of key genes in PI3K/AKT pathway and apoptosis pathway in HCC cells after MenSC treatment. The 5-hmC level of PIK3CD in enhancers was significantly decreased and the 5-mC level in PIK3R2 at enhancers was obviously increased after MenSC coculture. The 5-hmC level in TNFRSF1A and CASP7 at promoters were significantly enhanced. (PDF 1220 kb)

## Abbreviations

5-hmC: 5-Hydroxymethylcytosine
5-mC: 5-Methylcytosine
DHMR: Differentially hydroxymethylated region
DMR: Differentially methylated region
DNMT: DNA methyltransferases
HCC: Hepatocellular carcinoma
hMeDIP-Seq: Hydroxymethylated DNA immunoprecipitation sequencing
MeDIP-Seq: Methylated DNA immune-precipitation sequencing
MenSC: Menstrual blood-derived mesenchymal stem cell
MSC: Mesenchymal stem cell
TET: Tet methylcytosine dioxygenase
