# Supplementary material for: Genome-wide DNA methylation and hydroxymethylation analysis reveal human menstrual blood-derived stem cells inhibit hepatocellular carcinoma growth through oncogenic pathway suppression via regulating 5-hmC in enhancer elements
Source: Stem Cell Res Ther. 2019 May 31;10:151. doi: 10.1186/s13287-019-1243-8 (PMC6544940; doi:10.1186/s13287-019-1243-8)
Supplement: Supplementary file 2 — Figure S1. Alterations of 5-hmC and 5-mC of key genes in PI3K/AKT pathway and apoptosis pathway in HCC cells after MenSC treatment. The 5-hmC level of PIK3CD in enhancers was significantly decreased and the 5-mC level in PIK3R2 at enhancers was obviously increased after MenSC coculture. The 5-hmC level in TNFRSF1A and CASP7 at promoters were significantly enhanced. (PDF 1220 kb) [file 13287_2019_1243_MOESM2_ESM.pdf]

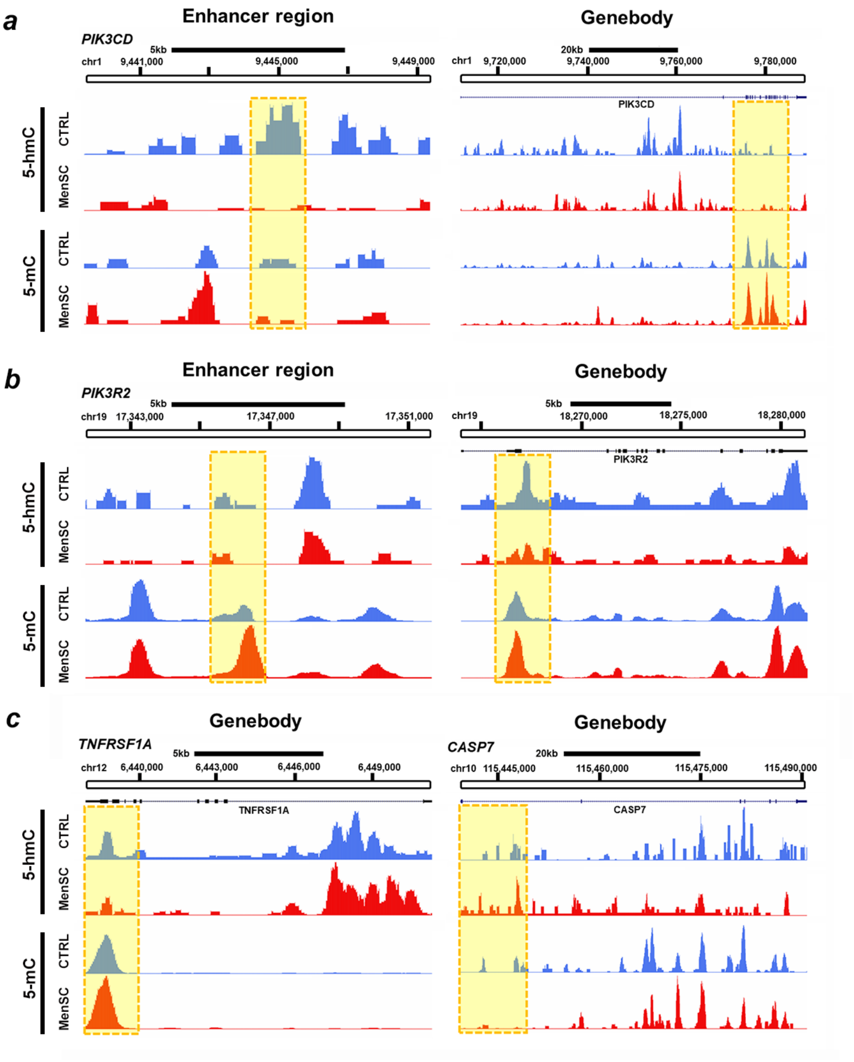

Fig. S1 Alterations of 5-hmC and 5-mC in the gene body and regulatory regions of key genes in PI3K/AKT pathway and apoptosis pathway in HCC cells after MenSC treatment.
